# Prevalence of *Helicobacter pylori* genotypes: *cagA, vacA (m1), vacA (s1), babA2, dupA, iceA1, oipA* and their association with gastrointestinal diseases. A cross-sectional study in Quito-Ecuador

**DOI:** 10.1186/s12876-023-02838-9

**Published:** 2023-06-06

**Authors:** Santiago Bustos-Fraga, Marco Salinas-Pinta, Yosselin Vicuña-Almeida, Ricardo Brandt de Oliveira, Lucy Baldeón-Rojas

**Affiliations:** 1 Departamento de Gastroenterología Clínica, Hospital General Docente de Calderón, Quito, Ecuador; 2 Facultad de Ciencias Médicas, Universidad Central del Ecuador, Quito, Ecuador; 3 Instituto de Investigación en Biomedicina, Universidad Central del Ecuador, Quito, Ecuador; 4 Facultad de Medicina de Ribeirão Preto, Universidad de São Paulo, São Paulo, Brasil

**Keywords:** *Helicobacter pylori*, Prevalence, Gastrointestinal diseases, Polymerase chain reaction

## Abstract

**Background:**

The most prevalent stomach infection in the world is caused by *Helicobacter pylori* (*H. pylori*). Several pathogenicity genes, including *cagA, vacA, babA2, dupA, iceA,* and *oipA*, are associated with an increased risk of gastrointestinal disease such as peptic ulcer and stomach cancer. This research aims to determine the prevalence of different *H. pylori* genotypes and correlate their risk in the development of gastrointestinal diseases in the Ecuadorian population.

**Methods:**

A cross-sectional research of 225 patients at the Calderón Hospital in Quito, Ecuador, was conducted. End point PCRs were run to determine the presence of *16S rRNA, cagA, vacA (m1), vacA (s1), babA2, dupA, iceA1, and oipA* virulence genes. Chi-square test, odds ratios (OR) and 95% confidence intervals (CI) were utilized for the statistical analysis.

**Results:**

*H. pylori* infection was present in 62.7% of people. Peptic ulcers were seen in 22.2% and malignant lesions in 3.6% of patients. Genes *oipA* (93.6%), *vacA (s1)* (70.9%), and *babA2* (70.2%) were the most prevalent. *cagA/vacA (s1m1)* and *cagA/oipA (s1m1)* combinations were found in 31.2% and 22.7% of the cases, respectively. Acute inflammation has a significant correlation with the genes *cagA* (OR = 4.96 95% CI: 1.1–22.41), *babA2* (OR = 2.78 95% CI: 1.06–7.3), and the *cagA/oipA* combination (OR = 4.78, 95% CI: 1.06–21.62). Follicular hyperplasia was associated with *iceA1* (OR = 3.13; 95% CI: 1.2–8.16), *babA2* (OR = 2.56; 95% CI: 1.14–5.77), *cagA* (OR = 2.19; 95% CI: 1.06–4.52), and the *cagA/oipA* combination (OR = 2.32, 95% CI: 1.12–4.84). The *vacA (m1)* and *vacA (s1m1)* genes were associated with gastric intestinal metaplasia (OR = 2.71 95% CI: 1.17–6.29) (OR = 2.33 95% CI: 1.03–5.24). Finally, we showed that *cagA/vacA (s1m1)* gene combination increased the risk of duodenal ulcer development (OR = 2.89, 95% CI 1.10–7.58).

**Conclusion:**

This study makes a significant contribution by offering genotypic information regarding *H. pylori* infection. The presence of several *H. pylori* genes was associated with the onset of gastrointestinal illness in the Ecuadorian population.

## Introduction

*Helicobacter pylori (H. pylori)* is the microorganism responsible for the most common stomach infection worldwide. Currently, its prevalence is higher in Africa, South America, and Western Asia and is related to an increased incidence of gastric cancer [1]. In Latin America and Caribbean, *H. pylori* infection affects 48.36% of children and adolescents, reaching a prevalence of 69.26% in adulthood [2]. The high rate of infection in these countries is related to a high percentage of chronic complications, becoming a health problem of transcendental importance [1].

*H. pylori* infection and the emergence of gastric and duodenal ulcers as well as gastric adenocarcinoma are closely related. The exact mechanisms are still unknown since they are long-term processes influenced by dietary factors, the host's genetic vulnerability, environment influence and the virulence of the *H. pylori* strains [3]. Seven distinct strains of *H. pylori* can be distinguished based on their geographic origin according to the structure's high genetic variability [4, 5]. European, Australian, Asian, East Asian, North African, South African, and West African. The Colombian Andes have been revealed to have the European genotype, which was probably brought from that continent thousands of years ago [6].

The virulence of *H. pylori* is correlated with several pathogenicity genes *(cagA, vacA, babA2, dupA, iceA, and oipA)*, whose proteins take part in colonization procedures, immune response evasion, and the presence of these genes has been considered to be a predictor of severe clinical consequences [7]. For instance, cytotoxin associated with gene A (CagA) can alter the cell cycle, induce apoptosis, and cause the release of interleukins that promote inflammation. The epigenetic alterations brought on by this toxin, such as histone hypermethylation, can also result in the downregulation of tumor suppressor genes [8]. It appears that the EPIYA-A, EPIYA-B, EPIYA-C, and EPIYA-D motifs in its C-terminal region are associated for virulence and carcinogenesis [9].

Gastric cells undergo several changes as a result of the vacuolating cytotoxin linked to gene A (VacA), including the formation of cytoplasmic vacuoles, permeabilization of the plasma membrane, mitochondrial fragmentation, and activation of kinase enzymes [10]. VacA presents three polymorphic areas, the signal region *(s)*, intermediate region *(i)*, and middle region *(m)*. It has been shown that the *vacA s1m1* genotype is the most hazardous due to its high production of toxins that result in the release of inflammatory chemicals that alter the gastric mucosa and increase the risk of gastric adenocarcinoma [11].

The *H. pylori* outer membrane contains the blood group antigen-binding adhesin (BabA), which is encoded by the *babA2* gene. BabA can detect the highly expressed Lewis b blood group antigen (Leb) in gastric cells [12]. BabA contributes to CagA's translocation via the type IV secretion system and signal transduction (T4SS), which causes the stomach mucosa to become severely inflamed. BabA has been linked to a higher incidence of chronic gastritis, distal gastric cancer, and peptic ulcers [13].

Duodenal ulcer risk has been linked to the gene *dupA* due to increased mucosal inflammation, neutrophil infiltration, and increased interleukin-8 (IL-8) expression in the antrum. Additionally, it has been linked to an increased risk of atrophy, gastric cancer, and intestinal metaplasia [14].

One candidate for a marker for peptic ulcer propensity is the A gene induced by contact with the epithelium (*iceA*). Although they share a location in the bacterial genome, their two variations, *iceA1,* and *iceA2*, are unrelated. Increased levels of IL-8 in the stomach mucosa have been related to peptic ulcer disease caused by infection with the *iceA1* strain [15].

The *oipA* gene encodes an inflammatory outer membrane protein with activity for bacterial adhesion to the mucosa. Independent of other virulence factors, infection with the active OipA strain has been linked to an increased risk of duodenal ulcer [16].

Gastric cancer was the seventh leading cause of death in Ecuador in 2019 and was most prevalent in people between the ages of 30 and 64 [17]. Although *H. pylori* infection is a public health issue, in Ecuador, there is no research supporting the pathogenic role of the *H. pylori* virulence genes in the development of gastrointestinal diseases. Therefore, the purpose of this research is to determine the prevalence of different *H. pylori* genotypes and correlate their presence with the probability of developing gastrointestinal clinical and histological changes.

## Materials and methods

### Study design

A total of 225 patients who underwent gastrointestinal endoscopies at the Calderón Hospital in Quito, Ecuador, between August 2020 and February 2021 were included in this observational, cross-sectional study.

### Inclusion and exclusion criteria

**Fig. 1:**
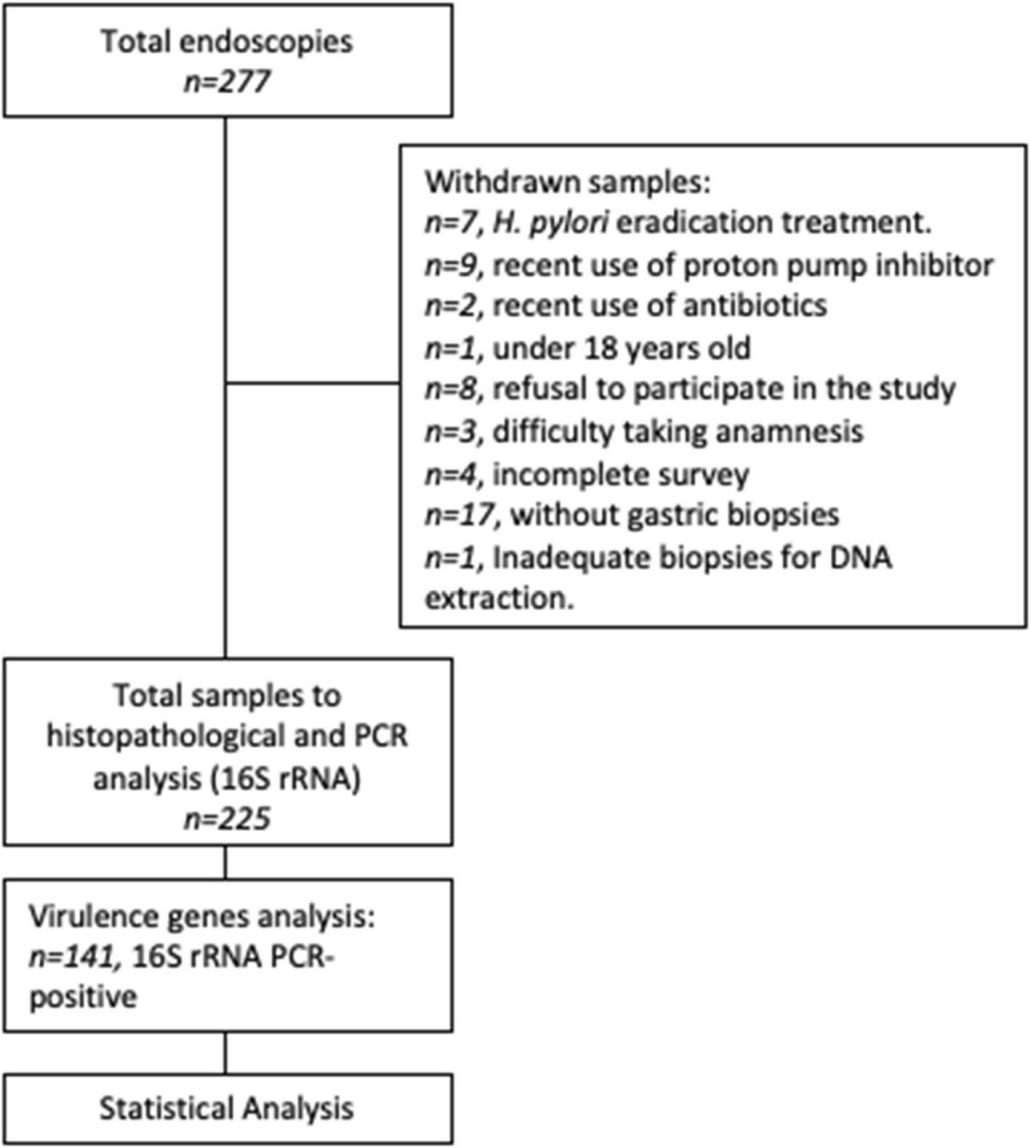
Chart flow with the summary of the methodology used in this study

The included patients were adults over the age of 18, had not received any antibiotics or proton pump inhibitors at least one month before the endoscopic procedure, nor had they received treatment to eradicate *H. pylori* during the previous 12 months (see Fig. 1).

### Data collection

The sociodemographic and clinical information were obtained from the patients who voluntarily accepted participate in the study. Gastric biopsies of the antrum, body, and incisura angularis were taken during the endoscopic examination for histological analysis in the pathological anatomy laboratory of the Calderón Hospital, following the modified Sydney protocol [18]. The Central University of Ecuador's "Dr. Rodrigo Fierro Benitez" Biomedicine Research Institute conducted molecular diagnostics and genotyping. The study was approved by the Ethics Committee for Research in Human Beings of the Central University of Ecuador (0121-SEISH-UCE-20) and the National Directorate of Health Intelligence of the Ministry of Public Health of Ecuador (MSP-DIS 2020–0135-O). An informed consent was obtained from the participants. Legally Authorized Representatives of illiterate participants provided informed consent for the study. In this case, the details of the study were explained verbally in the company of the legally authorized representative of the illiterate participants and the participant's fingerprint was placed on the informed consent.

### Genotyping

Following the manufacturer's instructions, DNA was extracted from the antrum biopsy using the PureLink Genomic DNA kit (Cat. K1820-02, Invitrogen). The samples were eluted in 50 uL of buffer, and Nanodrop One was used to assessing their quality. The DNA samples were kept at -40 °C in concentrations of 30 ng/L. Using primers designed for the hypervariable zone (V4-V9) of the 16SrRNA region, polymerase chain reaction (PCR) was used to detect the presence of *H. pylori*. The *cagA, vacA (m1), vacA (s1), babA2, iceA1, dupA,* and *oipA* genes were subsequently tested in samples that had previously tested positive for *H. pylori* (see Table 1).

**Table 1:** Primers sequences

Genes		Sequences (5´—3´)	Fragment (bp)	Reference
*16S rRNA H,pylori*	Forward	GCGGGATAGTCAGTCAGGTG	706	Valenzuela, 2016 [19]
	Reverse	AAGATTGGCTCCACTTCGCA		
*cagA*	Forward	GTTGATAACGCTGTCGCTTC	350	Chattopadhyay et al., 2004 [20]
	Reverse	GGGTTGTATGATATTTTCCATAA		
*vacA (m1)*	Forward	GGTCAAAATGCGGTCATGG	290	Atherton et al., 1995 [21]
	Reverse	CCATTGGTACCTGTAGAAAC		
*vacA (s1)*	Forward	CGCATCAACACTAACGCTGA	206	Delgado, 2018 [22]
	Reverse	GCATAACCGCCCACTTGATT		
*iceA1*	Forward	ATAAGCGGTTGGAGTTTGCG	220	Delgado, 2018 [22]
	Reverse	TATTCCTGCACCAACTCCCC		
*dupA*	Forward	GGATTTACCGCTTCCTGTGC	228	Delgado, 2018 [22]
	Reverse	GGATTTACCGCTTCCTGTGC		
*babA2*	Forward	AAAGATGATCACAGACGCGC	269	Delgado, 2018 [22]
	Reverse	TTGAGGGGTTGTTGCATGTG		
*oipA*	Forward	CAAGCGCTTAACAGATAGGC	430	Torres et al., 2014 [23]
	Reverse	AAGGCGTTTTCTGCTGAAGC		

The GoTaq Green Master Mix (2X) (Cat. M7123, Promega), 0.2 to 0.5 uM of each primer, and 1 μL of DNA were combined to prepare the PCR reactions with a final volume of 25 µL per reaction. The parameters used for PCR amplification were similar to the references in Table 1. The thermocycler used for the amplification was a GeneAmp System 2700. (Applied Biosystem, USA). The PCR results were observed using electrophoresis in 1.5% TBE 1X agarose gels, which were stained with Sybr Safe and ran for 60 min at 100 V to determine the size of the bands.

### Statistical analysis

Data are presented as total numbers and percentages, as appropriate. The chi-square test was used to analyze significant differences between the frequencies of sociodemographic variables (sex, age, education and ethnicities) and the result of *H. pylori* diagnostic and clinical/histopathological characteristics. Odds ratios (OR) and 95% confidence intervals (CI) were calculated to assess the association between gastric diseases and the presence of *H. pylori* genes (*cagA, vacA (m1), vacA (s1), babA2, iceA1, dupA, oipA*). Statistics were considered to be significant at *p* < 0.05. Software IBM, SPSS version 23 was utilized for the statistical analysis.

## Results

### Sociodemographic characteristics

The study included 225 patients who met the inclusion criteria. Histological diagnostic revealed *H. pylori* infection in 58.7% of the cases, in contrast to PCR where 62.7% of the participants confirmed the infection. Using the PCR results, we identified significant association between age and *H. pylori* infection, being more frequent in those under 40 years old. Gender, ethnicity, low level (illiterate, elementary and high school) and high level (University) of education did not appear to be significant factors (see Table 2).

**Table 2:** Sociodemographic characteristics of the patients

Variable	All n(%)^†^	*H. pylori*-negative n(%)	*H. pylori*-positive n(%)	*p*-value^‡^
All	225(100)	84(37.3)	141(62.7)	
**Sex**				
Female	114(50.7)	40(35.1)	74(64.9)	0.480
Male	111(49.3)	44(39.6)	67(60.4)	
**Age (years)**				
< 40	72(32.0)	20(27.8)	**52(72.2)**	**0.019**
40–50	41(18.2)	12(29.3)	29(70.7)	
> 50	112(49.8)	52(46.4)	60(53.6)	
**Education**				
Low level	193(85.8)	72(37.3)	121(62.7)	0.983
High level	32(14.2)	12(37.5)	20(62.5)	
**Ethnicities**^**§**^				
Mestizo	208(97.7)	80(38.5)	128(61.5)	0.579
Indigenous	2(0.9)	1(50.0)	1(50.0)	
Afro-American	3(1.4)	2(66.7)	1(33.3)	

Abbreviations: *H. pylori (Helicobacter pylori)*

^†^n(%), represents the number (percentage) of patients with the conditions

^‡^Chi-square test was performed to determine the differences between sociodemographic variables and patients with a positive result for *H. pylori*. Bold values denote a statistically significant result at the *p* < 0.05 level

^§^Ethnicities variable included *n* = 213(100%), the remaining 12 did not answer the question

### Clinical and histological features

The endoscopic examination revealed that 22.2% of the patients had peptic ulcers (gastric or duodenal), which were more common in men (*p* = 0.042), in adults over 50 years (*p* = 0.034), and people with low level of education (*p* = 0.019). In particular, stomach ulcers occurred in 11.6% of patients, and duodenal ulcers in 13.8% (see Table 3).

**Table 3:** Clinical and Histopathological characteristics of the patients

Variable	Clinical Characteristics						Histopathological characteristics							
	**Peptic ulcer n(%)**^**†**^	***p*-value**^**‡**^	**Gastric ulcer n(%)**	***p*** **-value**	**Duodenal ulcer n(%)**	***p*** **-value**	**Lymphoid follicular hyperplasia n(%)**	***p*** **-value**	**Gastric atrophy n(%)**	***p*** **-value**	**Gastric intestinal metaplasia n(%)**	***p*** **-value**	**Gastric cancer n(%)**	***p*** **-value**
All	50(22.2)		26(11.6)		31(13.8)		71(31.6)		138(61.3)		65(28.9)		8(3.6)	
**Sex**														
Female	19(16.7)	**0.042**	13(11.4)	0.942	8(7.0)	**0.003**	40(35.1)	0.248	71(62.3)	0.288	35(30.7)	0.543	4(3.5)	0.969
Male	**31(27.9)**		13(11.7)		**23(20.7)**		31(27.9)		67(60.4)		30(27.0)		4(3.6)	
**Age (years)**														
< 40	11(15.3)	**0.034**	4(5.6)	**0.013**	8(11.1)	0.378	**36(50.0)**	** < 0.001**	33(45.8)	**0.011**	9(12.5)	**0.001**	1(1.4)	0.334
40–50	6(14.6)		2(4.9)		4(9.8)		15(36.6)		26(63.4)		13(37.1)		1(2.4)	
> 50	**33(29.5)**		**20(17.9)**		19(17.0)		20(17.9)		**79(70.5)**		**43(38.4)**		6(5.4)	
**Education**														
Low Level	**48(24.9)**	**0.019**	**26(13.5)**	**0.027**	29(15.0)	0.182	56(29.0)	**0.044**	120(62.2)	0.548	**61(31.6)**	**0.027**	7(3.6)	0.887
High Level	2(6.3)		0(0.0)		2(6.3)		**15(46.9)**		18(56.3)		4(12.5)		1(3.1)	
**Ethnicities**^**§**^														
Mestizo	43(20.7)	0.097	23(11.1)	0.118	26(12.5)	0.484	66(31.7)	0.628	132(63.5)	0.209	61(29.3)	0.808	6(2.9)	**0.013**
Indigenous	1(50.0)		1(50.0)		0(0.0)		0(0.0)		1(50.0)		1(50.0)		0(0.0)	
Afro-American	2(66.7)		1(33.3)		1(33.3)		1(33.3)		1(33.3)		1(33.3)		**1(33.3)**	

^†^n(%), represents the number (percentage) of patients with the conditions

^‡^All *p*-values were performed by chi-square test to determine the differences between sociodemographic variables and clinical/histopathological outcomes. Bold values denote a statistically significant result at the *p* < 0.05 level

^§^Ethnicities variable included *n* = 213(100), the remaining 12 did not answer the question

74.7% of the patients had acute gastritis, and neither gender, age, education level, nor ethnicity showed any significant differences. In 97.8% of the patients, the histological diagnostic of chronic gastritis was established; the frequency was higher in the indigenous population (*p* < 0.001) (data not shown). A higher prevalence of lymphoid follicular hyperplasia was seen in patients under 40 (*p* < 0.001) and in those with a high level of education (*p* = 0.044), accounting for 31.6% of the cases. In 61.3% of the cases, gastric atrophy was identified; it was significantly prevalent among individuals over 50 years (*p* = 0.011). Intestinal metaplasia was detected in 28.9% of cases, the elderly individuals exhibiting a greater prevalence (*p* = 0.001). Gastric cancer was diagnosed in 3.6% of the cases, showing significant differences in Afro-American ethnicity (*p* = 0.013) (see Table 3). Half of the cases with stomach cancer were revealed to have malignant lesions of Borrmann type 3 and the other half to have high-grade adenomas.

### *H. pylori* genotypes and clinical characteristics

Greater prevalence was seen for the genes *oipA* (93.6%), *vacA (s1)* (70.9%), and *babA2* (70.2%). *cagA, vacA (m1), vacA (s1m1), iceA1*, *dupA* and the combination *cagA/oipA* and *cagA/vacA, (s1m1)* were presented in less than 50% of the patients (see Table 4). The *cagA* gene was more common in adults between the ages of 40 and 50 (31.9%; *p* = 0.033) (data not shown). Moreover, we found that the *cagA/vacA (s1m1)* gene combination increased the risk of duodenal ulcer development (OR = 2.89, 95% CI 1.10–7.58). On the other hand, the presence of the genes *cagA* (OR = 0.22, 95% CI 0.05–0.98) and *babA2* (OR = 0.25, 95% CI 0.09–0.67) were linked to a lower risk of developing stomach ulcers (see Table 4).

**Table 4:** Association of clinical and histopathological characteristics with *H. pylori* virulence genes

Genes	Prevalence	Clinical Characteristics		Histopathological Characteristics			
		**Gastric Ulcer**	**Duodenal Ulcer**	**Acute inflammation**	**Lymphoid follicular hyperplasia**	**Gastric Atrophy**	**Gastric intestinal metaplasia**
	**(%)**	**OR (95%CI)**					
*cagA*	31.9	**0.22 (0.05–0.98)**	1.60 (0.63–4.07)	**4.96 (1.10–22.41)**	**2.19 (1.06–4.52)**	1.02 (0.45–2.34)	1.23 (0.53–2.85)
*vacA (m1)*	48.9	0.57 (0.21–1.53)	1.31 (0.52–3.25)	2.53 (0.91–7.03)	1.21 (0.61–2.39)	1.10 (0.50–2.39)	**2.71 (1.17–6.30)**
*vacA (s1)*	70.9	0.87 (0.31–2.48)	0.86 (0.32–2.29)	1.38 (0.51–3.75)	1.29 (0.60–2.75)	1.80 (0.77–4.19)	1.53 (0.60–3.90)
*vacA (s1m1)*	41.8	0.60 (0.21–1.69)	1.48 (0.59–3.68)	2.42 (0.82–7.06)	1.34 (0.68–2.67)	1.02 (0.47–2.21)	**2.33 (1.03–5.24)**
*iceA1*	14.9	0.28 (0.04–2.25)	1.89 (0.61–5.85)	3.76 (0.48–29.74)	**3.13 (1.20–8.16)**	**0.33 (0.12–0.91)**	0.55 (0.15–2.00)
*dupA*	48.9	1.96 (0.72–5.30)	1.63 (0.65–4.09)	0.59 (0.23–1.56)	1.07 (0.54–2.11)	0.76 (0.35–1.66)	1.90 (0.84–4.29)
*babA2*	70.2	**0.25 (0.09–0.67)**	2.11 (0.67–6.67)	**2.78 (1.06–7.30)**	**2.56 (1.14–5.77)**	0.43 (0.17–1.09)	0.86 (0.37–2.03)
*oipA*	93.6	1.26 (0.15–10.71)	1.51 (0.18–12.74)	1.81 (0.35–9.41)	-	2.36 (0.56–10.02)	2.35 (0.28–19.57)
*cagA/oipA*	31.2	0.22 (0.05–1.02)	1.66 (0.65–4.24)	**4.78 (1.06–21.62)**	**2.32 (1.12–4.84)**	1.16 (0.50–2.69)	1.28 (0.55–2.98)
*cagA/vacA (s1m1)*	22.7	0.36 (0.08–1.65)	**2.89 (1.10–7.58)**	-	2.21 (0.99–4.91)	1.00 (0.41–2.48)	0.99 (0.38–2.57)

Abbreviations: *H. pylori (Helicobacter pylori)*

*OR* Odds ratios was performed to determine the association between gastric pathologies and *H. pylori* virulence genes

%, represent the percentage of each gene present in the patients

*95% CI* 95% Confidence interval

Bold values denote a statistically significant result at the p < 0.05 level

### *H. pylori* genotypes and histopathological characteristics

The presence of the genes *cagA* (OR = 4.96, 95% CI 1.10–22.41), *babA2* (OR = 2.78, 95% CI 1.06–7.30), and the combination of *cagA/oipA* genes (OR = 4.78, 95% CI 1.06–21.62) were found to increase the risk of developing acute gastric inflammation. In addition, we found that the genes *cagA* (OR = 2.19, 95% CI 1.06–4.52), *iceA1* (OR = 3.13, 95% CI 1.20–8.16), *babA2* (OR = 2.56, 95% CI 1.14–5.77), and the *cagA/oipA* combination (OR = 2.32, 95% CI 1.12–4.84) all increased the likelihood of having lymphoid follicular hyperplasia (see Table 4).

Finally, the odds of developing gastric intestinal metaplasia were considerably enhanced by the presence of the genes *vacA (m1)* (OR = 2.71, 95% CI 1.17–6.30) and *vacA (s1m1)* (OR = 2.33, 95% CI 1.03–5.24), while the odds of developing gastric atrophy were significantly decreased by the presence of *iceA1* (OR = 0.33, 95% CI 0.12–0.91) (see Table 4).

## Discussion

In this study, the prevalence of *H. pylori* infection was 58.7% when determined by histology and 62.7% when determined by PCR. Histology allows direct visualization of the bacteria and is recommended for primary diagnosis if upper endoscopy is required. PCR test offer comparable specificity and sensitivity to the histology test [24]. Previous studies conducted in Quito, Ecuador, found low prevalence rates of 40.2% and 42.4%, respectively [25, 26]. As in other studies, no gender-related differences were discovered. Moreover, our study did not find a link between *H. pylori* infection and low levels of education, in contrast to other studies that found such a correlation [27]. Importantly, we described a negative correlation between age and the prevalence of *H. pylori*; as a result, patients under the age of 40 had a higher frequency than those over the age of 50. This finding disagrees with a systematic review done in China that found that the prevalence increases with age [28]. This observation may be explained by the high rates of gastric atrophy and metaplasia also seen in our study, since these conditions reduce the stomach's bacterial burden due to the lack of sustenance [29]. Therefore, the early onset of gastric atrophy may account for the low incidence of *H. pylori* infection in elderly individuals.

Several studies have shown a link between *H. pylori* infection and either acute or chronic gastritis [30]. In the current investigation, we were able to confirm that patients who had both acute and chronic inflammation were significantly more likely to have *H. pylori*. (74.7% and 97.8%, respectively). Additionally, we identified that individuals over 50 years of age had higher rates of peptic ulcer, atrophy, and stomach metaplasia. This finding was consistent with earlier research done in Peru and China [31, 32]. It has been noted that intestinal metaplasia and a reduction in gastric secretion are related to the absence of *H. pylori* infection [33]. We confirmed that *H. pylori* infection had a protective effect against the development of gastric intestine metaplasia (OR 0.41; 95% CI: 0.23–0.75).

It is significant to note that MALT (mucosa-associated lymphoid tissue) lymphoma can develop as a result of the host's inflammatory immunological response to *H. pylori* infection. We found that lymphoid follicular hyperplasia was prevalent in this study at a frequency of 31.6%, which is consistent with other researchers' findings [34].

There is great geographic heterogeneity in the prevalence of *H. pylori* genotypes, which is linked to clinical manifestations that can be severe in some areas but less harmful or even non-aggressive in others [7]. A pathogenic strain's presence alone is insufficient to start a pathological process. For instance, several African cultures with a 100% prevalence of *H. pylori* infection and highly virulent strain (hspsafrica, hspwafrica, hpafrica2, hpneafrica) had low rates of stomach cancer. A condition referred to as " The African paradox" [35].

Due to their high virulence, the *cagA* and *vacA* genes are among the most studied. In our study, the *cagA* genotype, which is a marker of the existence and activity of the pathogenicity island (cagPAI), was uncommon (31.9%). The frequency of this gene varies greatly across the world between 60%—80% [36, 37]. We found a significant correlation between the *cagA* genotype and the emergence of acute gastric inflammation in this cohort (OR 4.96; 95% CI: 1.10—22.41). We also present evidence-linking *cagA* to stomach lymphoid follicular hyperplasia, which has the potential to develop into MALT lymphoma over time (OR 2.19; 95% CI: 1.06—4.52). Furthermore, numerous studies have shown a link between *cagA* and acid peptic disease, chronic atrophic gastritis, and stomach cancer [38, 39]. However, we did not find evidence of this relationship; we even found a protective effect of the presence of *cagA* gene and the development of gastric ulcer (OR 0.21; 95% CI: 0.04–0.98). This data might be related to the low prevalence found or the local strains' lower virulence. It is intriguing to learn that some *H. pylori* genes are apparently naturally disappearing from western populations, especially Cag-A positive strains, which are selectively eliminated because of their link to peptic ulcers [40].

In our research, the presence of the *H. pylori vacA s1m1* genotype was 41.8%, which is quite comparable to the 42% reported by Shetty et al. in India [37]. Geographically, the *m1* genotype is more common among the population of Africa and the *m2* genotypes are equally distributed in Latin America and Europe [41]. The *s1m1* genotype is crucial because of the large levels of toxin that this genotype secrets, which have been associated with serious lesions in the stomach. In this study, the development of gastric-intestinal metaplasia was linked to the *vacA m1* and *vacA s1m1* genotypes (OR 2.71, 95% CI: 1.17—6.30) (OR 2.33, 95% CI: 1.03—5.24). These findings are in line with research done in populations from Brazil and the Middle East, which links *vacA s1* or *m1* to the development of stomach cancer [38, 42]. 

*iceA1* genotype has been linked to the onset of peptic ulcers due to the gene's ability to cause excessive production of the pro-inflammatory cytokine IL-8 [43]. When compared to studies from other nations, where its frequency is 53.3%, our study's prevalence of *iceA1* (14.9%) is considerably low [36]. In the Brazilian population this gene was linked to the development of gastritis but not peptic ulcers [44]. In China and Egypt however, it was associated with peptic ulcers [15, 45]. In this research, we found that the *iceA1* gene was associated with lymphoid follicular hyperplasia (OR 3.13; 95% CI: 1.20—8.16) rather than gastritis or peptic ulcer development.

The prevalence of the *dupA* gene was 48.9% in this study, which is comparable to the prevalence estimates in Belgium (43.7%) and South Africa (53.4%) [46]. Despite the *dupA* gene being suggested as a marker for peptic ulcers, our research, like that of other authors, was unable to validate this association [47].

Our study found a prevalence of *babA2* of 70.2%, comparable to that of an Iranian study [48], but lower than that of Thailand (92%) [49]. In Western nations, but not in Asian nations, *babA* gene is linked to an increased risk of acid peptic illness [50]. Intriguingly, babA2 in our study showed a protective effect against the occurrence of gastric ulcers (OR 0.24; 95% CI: 0, 09–0, 67), but on the other hand, it was associated with the occurrence of acute inflammation (OR 2.78; 95% CI: 1.06—7.30) and lymphoid follicular hyperplasia (OR 2.56, 95% CI: 1.14—5.77). This discovery supports the observation made by Chen et al. [50].

Finally, our study revealed a high prevalence of the *oipA* gene (93.6%), similar to studies carried out in Venezuela (93.8%) and China (88.1%—100%) [23, 51]. The *oipA* genotype is significantly associated with an increased risk of acid peptic disease (OR = 3.97) and gastric cancer (OR = 2.43) [52, 53]. However, unlike several studies conducted internationally, we did not show this association.

The results of global genomic investigations are inconsistent and controversial. This study is another example of the genetic diversity of *H. pylori* observed by the different prevalences discovered. Which may be a reflection of demographic diversity, *H. pylori* strain-specific virulence factors, host genotype, and environmental factors such as diet [3].

## Limitations

Due to the fact that the current study was conducted in a single medical facility, we cannot rule out the possibility of selection bias. To acquire data that may be extrapolated to the Ecuadorian population, it is also important to conduct studies with a larger population.

Technically speaking, the possibility of false negative findings should be a key factor to take into account. Factors including inaccurate biopsy taking, low bacterial DNA concentrations, and PCR failures might result in erroneous results. In this investigation, all procedures were carried out in accordance with defined handling and transport guidelines. The isolated DNA's quality and concentration were confirmed. Both positive and negative controls were used in the 16S rRNA-PCR (samples with and without *H. pylori*, as determined by histology and later confirmed by PCR). And, for genotyping, a specific band for each gene and non-amplification of the NTC (Non-template control) were the parameters used to validate the results.

Additionally, in line with the argument of the validation of our results, the frequencies we discovered are comparable to those reported by other study groups. However, *H. pylori's* genetic diversity must be taken into consideration.

## Conclusion

This study presents the genotypic prevalence of *H. pylori* existing in Ecuadorian patients. The histological presence of acute gastric inflammation and follicular hyperplasia are associated to the genes *cagA, babA2*, *iceA1* and *cagA/oipA*. The presence of *vacA (m1)* and *vacA (s1m1)* increased the risk of intestinal metaplasia. Finally, *cagA/vacA (s1m1)* is related with duodenal ulcer. Our findings suggest that the clinical course of the disease is influenced by the host's immune response, the presence of *H. pylori* virulence genes, and environmental variables.

## Data Availability

All data generated or analyzed during this study are included in this published article.

## Abbreviations

*H. pylori*: *Helicobacter pylori*
CagA: Cytotoxin associated with gene A
VacA: Vacuolating cytotoxin linked to gene A
BabA: Blood group antigen-binding adhesin
*iceA*: A gene induced by contact with the epithelium
PCR: Polymerase chain reaction
OR: Odds ratios
CI: Confidence intervals
MALT: Mucosa-associated lymphoid tissue
cagPAI: Cag pathogenicity island
